# The Histopathological Finding of the Surgically Extracted Atypical Dome-Shaped Choroidal Osteoma

**DOI:** 10.1155/2017/2874823

**Published:** 2017-03-15

**Authors:** Hirona Bessho, Hisanori Imai, Atsushi Azumi

**Affiliations:** ^1^Department of Ophthalmology, Kobe Kaisei Hospital, Kobe 657-0068, Japan; ^2^Department of Organ Therapeutics, Division of Ophthalmology, Kobe University Graduate School of Medicine, Kobe 650-0017, Japan

## Abstract

*Purpose*. To report a case of atypical dome-shaped choroidal osteoma, which was diagnosed by histopathological finding of surgically extracted tumor.* Case Report*. A 35-year-old woman presented with visual field abnormality in the left eye (OS). Her best-corrected visual acuity with Landolt ring chart was 1.0 OS. The funduscopic examination revealed a yellowish dome-shaped choroidal tumor located in the temporal side of the macula with exudative retinal detachment. 25-gauge pars plana vitrectomy and the extraction of the tumor were performed for the definitive diagnosis.* Results*. As a result of histopathological finding from the extracted tumor, she was diagnosed with choroidal osteoma. 10 months after the last surgery, the BCVA is 0.7 OS. The tumor is not relapsed.* Conclusions*. We must keep in mind that choroidal osteoma can be one of the differential diagnoses for the dome-shaped choroidal tumor.

## 1. Introduction 

Choroidal osteoma is a rare benign intraocular tumor characterized by heterotopic bone of the choroid. Typical choroidal osteoma is slightly elevated from the peripapillary or macular choroid [1–5]. Usually, its characteristic funduscopic, computed tomography (CT), and B-scan ultrasonography findings are sufficient for a clinical diagnosis [6]. However, we face a particular diagnostic problem in case that the clinical and imaging findings are atypical because the biopsy and extraction of this tumor for definitive histopathological diagnosis are considerably difficult [6–8].

Here, we report a case of atypical dome-shaped choroidal osteoma diagnosed by a histopathological finding from the surgically extracted tumor tissue.

## 2. Case Report

**Figure 1:**
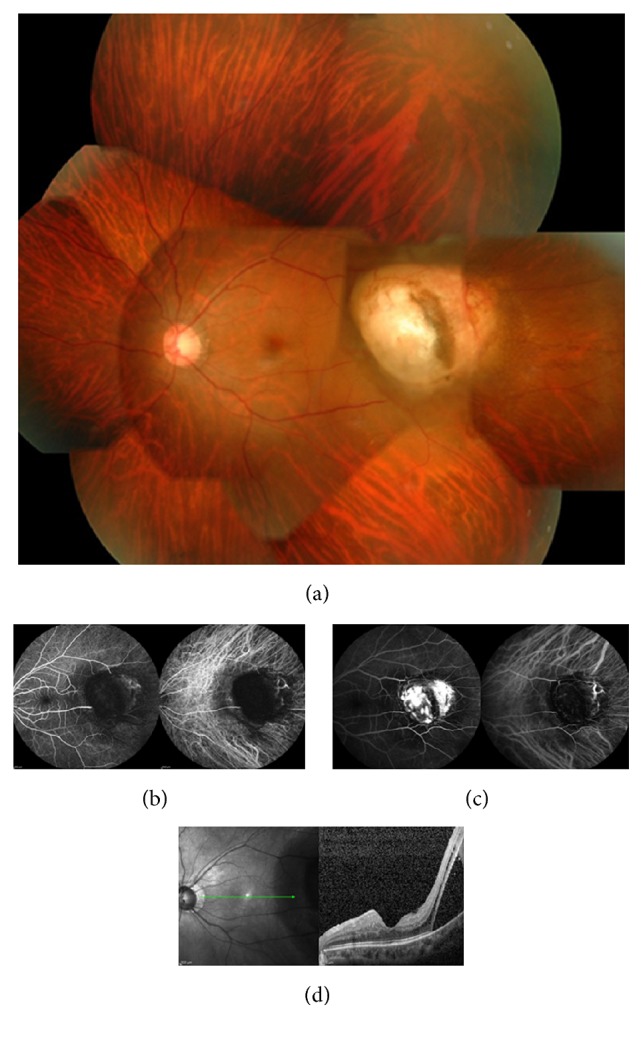
(a) A fundus examination revealed a yellowish dome-shaped choroidal tumor. (b) Early phase of the fluorescein (left) and indocyanine (right) angiography. (c) Late phase of the fluorescein (left) and indocyanine (right) angiography. The findings of angiographies revealed the pooling of the dye into the tumor. (d) The OCT finding of exudative retinal detachment surround the tumor.

**Figure 2:**
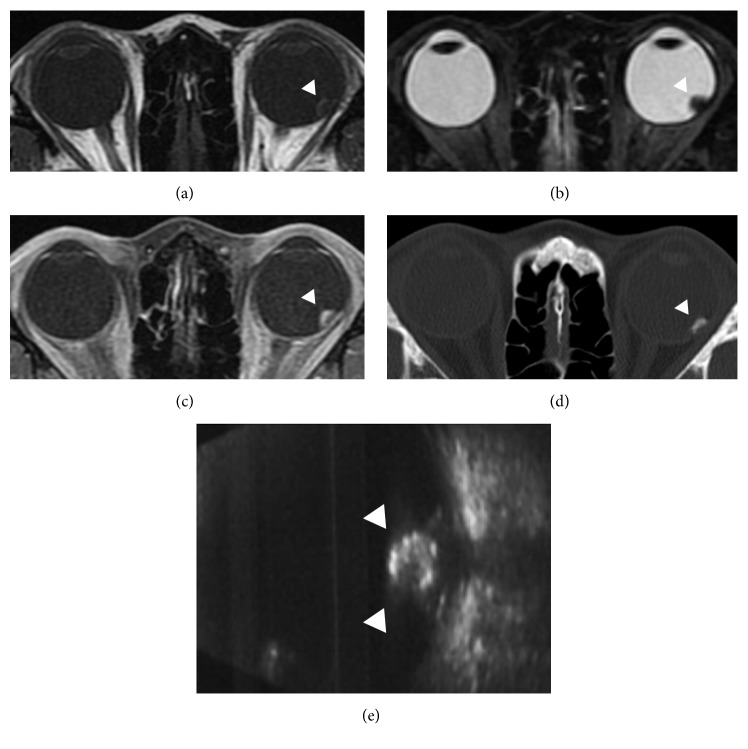
The tumor was depicted as high intensity on both T1-weighted (a) and gadolinium enhanced (c) MRI, low intensity on STIR MRI (b), and high density lesion on CT (d). The B-scan ultrasonography showed high internal reflectivity and acoustic shadowing (e) (arrow heads).

**Figure 3:**
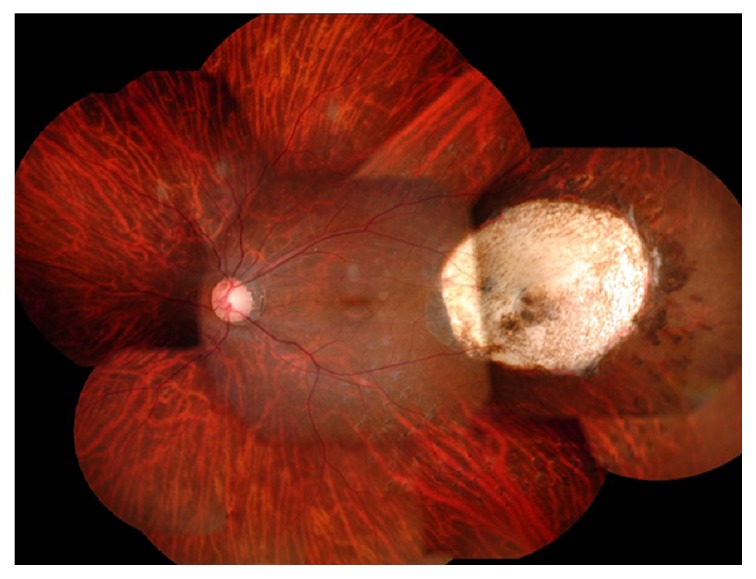
A fundus examination 10 months after the primary operation revealed bare sclera at the temporal side of the macula where the tumor was located.

A 35-year-old woman was referred to us with visual field abnormality in the left eye (OS). On examination, best-corrected visual acuity (BCVA) with Landolt ring chart was 1.0 in both eyes at the initial visit. The slit-lamp examination of the anterior segment was normal OS. A fundus examination revealed a yellowish dome-shaped choroidal tumor of approximately 4-disc diameters in size located to the temporal side of the macula with exudative retinal detachment OS (Figures 1(a) and 1(d)). The right-eye findings were unremarkable. Fluorescein and indocyanine angiography revealed the pooling of the dye into the tumor (Figures 1(b) and 1(c)). The tumor was depicted as high intensity on both T1-weighted (Figure 2(a)) and gadolinium enhanced (Figure 2(c)) magnetic resonance images (MRI), and low intensity on short-T1 inversion recovery (STIR) MRI (Figure 2(b)), with maximal dimensions of 5.3 × 5.7 × 6.0 mm. The tumor was depicted as high density lesion on CT (Figure 2(d)). B-scan ultrasonography showed high internal reflectivity and acoustic shadowing (Figure 2(e)). N-Isopropyl-p-123I-iodoamphetamine single photon emission computed tomography (SPECT) obtained at 24 hours after intravenous administration of 123I-IMP showed no positive area. The serum 5-S-cysteinyldopa (5-S-CD) level was high at 9.5 nmol/l (normal range 1.5–8.0 nmol/l). The findings of fundus, MRI, and SPECT and the value of serum 5-S-CD are compatible with an amelanotic malignant melanoma, but the results of other examinations suggested the tumor which is possibly calcified. For a confident diagnosis, we performed the extraction of the tumor under the written informed consent from the patient and her father. The procedure of the primary operation was listed below. Additional pars plana vitrectomy (PPV) for silicone oil removal and for epiretinal membrane removal and intraocular lens implantation were performed one and three months after the primary operation, respectively. 10 months after the last surgery, the BCVA is 0.7 OS. Choroidal osteoma is not relapsed (Figure 3).

## 3. Surgical Technique for the Primary PPV

Initially, we tried a diagnostic biopsy for the tumor using the standard 25-gauge three-port PPV.

But the biopsy was failed because the tumor was calcified and therefore unresectable. As the next best thing, we performed the extraction of the tumor. Briefly, retinectomy at the surface of the tumor was done after total vitrectomy was performed. 20-gauge pars plana sclerotomies were made by 20-gauge V-lances at 3 and 9 o'clock position. The tumor was chipped off from sclera by the V-lances and a 25-gauge intraocular scissor. The phacoemulsification was performed from 2.4 mm scleral tunnel and then the lens capsule was grasped and removed by 23-gauge capsulorhexis forceps from the corneal side port. The scleral tunnel was extended to 10 mm incision and the tumor was removed from this site. The vitreous cavity was filled with silicone oil at the end of the operation. The surgically excised tumor was fixed with 10% formalin until use (Supplemental Digital Content in Supplementary Material available online at https://doi.org/10.1155/2017/2874823).

## 4. Histopathological Analysis

**Figure 4:**
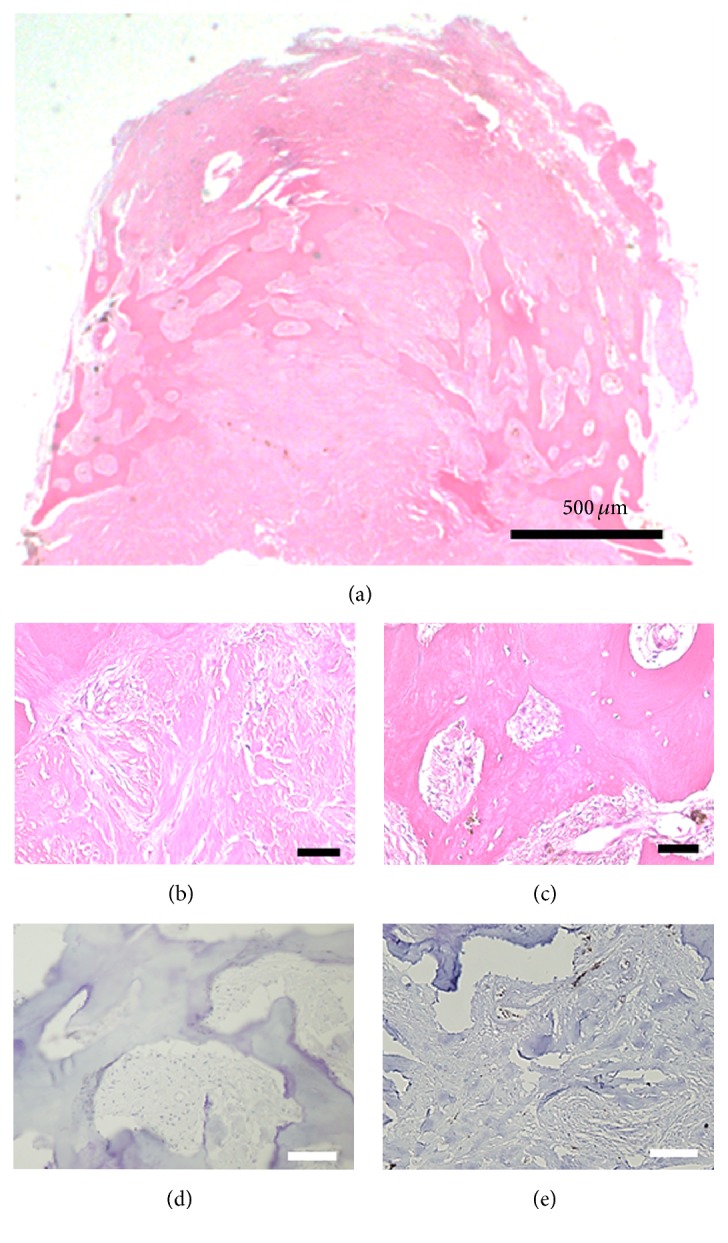
The histopathological finding of the surgically extracted tumor. (a) Hematoxylin-eosin staining of the whole image of extracted tumor. Magnified picture of part of (a) shows hyalinization (b) and bone formation (c). The immunostaining for epithelial membrane antigen (d) and glial fibrillary acidic protein (e) was negative (scale bar: black bar = 20 *μ*m; white bar = 50 *μ*m).

Several types of immunohistochemical staining were performed under the patient's writing informed consent. Hematoxylin-eosin staining highlighted the bone formation and hyalinization without osteoblasts (Figures 4(a), 4(b), and 4(c)). Several spindle cells infiltrated around the bone formation. Several pigment-having mononuclear cells were intermingled, suggesting retinal pigment epithelium. The immunostaining for epithelial membrane antigen (Figure 4(d)) and glial fibrillary acidic protein (Figure 4(e)) was negative. From these findings, we diagnosed this tumor as a choroidal osteoma.

## 5. Discussion

In our case, the clinical feature of the tumor was atypical for all differential diagnoses, including choroidal osteoma and amelanotic malignant melanoma, so it was extremely challenging to make a clinical diagnosis as choroidal osteoma using conventional noninvasive examinations. Additional histopathological confirmation of the diagnosis was desirable for differentiation of this calcified tumor from amelanotic malignant melanoma, since the management of intraocular tumors includes different treatment options depending on the pathogenesis [9].

Various intraocular biopsy methods have been proposed with different success rates and side effects [10–16]. In our case, we reluctantly performed the extraction of the tumor because the biopsy was difficult. As a result, we could make a definitive diagnosis and avoid unnecessary invasive treatments including enucleation, radiotherapy, and chemotherapy. However, we eventually needed two additional operations to maintain her visual function. So, we emphasize that the extraction of the tumor could be one of the considerable options when the conclusive diagnosis is needed, but these kinds of invasive techniques should be carefully performed because of concerns for iatrogenic vision-threatening ocular complications or extraocular seeding of tumor cells following tumor sampling [9, 13–18].

The histopathological finding of the choroidal osteoma from the living eye is extremely rare, because the morphological confirmation was typically performed after enucleation of the eye. In our case, we finally narrowed down the differential diagnosis to choroidal osteoma, retinal pigment epithelium metaplasia, and retinal astrocytoma. We excluded retinal pigment epithelium metaplasia and retinal astrocytoma because the immunostaining for epithelial membrane antigen and glial fibrillary acidic protein was negative, respectively. We believe that histopathological finding in our case could be one of the important references for the differential diagnosis of the atypical choroidal tumor confused with other entities with similar presentations.

In conclusion, we must keep in mind that choroidal osteoma can be one of the differential diagnoses for atypical dome-shaped yellowish choroidal tumor.

## Supplementary Material

We performed the extraction of the tumor. Briefly, endolaser photocoagulation was applied to surround the tumor and the retinectomy at the surface of the tumor was done after total vitrectomy was performed. 20-gauge pars plana sclerotomies were made by 20-gauge V-lances at 3 and 9 o'clock position. The tumor was chipped off from sclera by the V-lances and a 25-gauge intraocular scissor.
